# Simulation of Chordate Intron Evolution Using Randomly Generated and Mutated Base Sequences

**DOI:** 10.1177/1176934320903108

**Published:** 2020-01-29

**Authors:** Guang-Dong Wang, Yong Wang, Zhen Zeng, Jun-Ming Mao, Qin-Liu He, Qin Yao, Ke-Ping Chen

**Affiliations:** 1 Institute of Life Sciences, Jiangsu University, Zhenjiang, China; 2 School of Food and Biological Engineering, Jiangsu University, Zhenjiang, China

**Keywords:** Introns-early theory, introns-late theory, evolutionary model, mutation-and-deletion, mutation-and-insertion

## Abstract

Introns are well known for their high variation not only in length but also in base sequence. The evolution of intron sequences has aroused broad interest in the past decades. However, very little is known about the evolutionary pattern of introns due to the lack of efficient analytical method. In this study, we designed 2 evolutionary models, that is, mutation-and-deletion (MD) and mutation-and-insertion (MI), to simulate intron evolution using randomly generated and mutated bases by referencing to the phylogenetic tree constructed using 14 chordate introns from *TF4* (transcription factor–like protein 4) gene. A comparison of attributes between model-generated sequences and chordate introns showed that the MD model with proper parameter settings could generate sequences that have attributes matchable to chordate introns, whereas the MI model with any parameter settings failed in doing so. These data suggest that the surveyed chordate introns have evolved from a long ancestral sequence through gradual reduction in length. The established methodology provides an effective measure to study the evolutionary pattern of intron sequences from organisms of various taxonomic groups. (C++ scripts of MD and MI models are available upon request.)

## Introduction

Introns are nucleotide sequences interrupting the coding regions (exons) in a gene, which are frequently seen in eukaryotic protein-coding genes. The evolution of intron sequences has aroused broad interest in the past decades. However, 2 contrary hypotheses, namely, the “introns-early” versus the “introns-late” theories, put forward to explain the evolutionary mechanisms of introns are still under debate. Introns-late theory proposes that introns are an innovation of eukaryotes and intron gain has been a continuous process during the evolution of eukaryotes.^1,2^ This theory is supported by the facts that all current prokaryotic genes are free of spliceosomal introns, and intron number and length in eukaryotes increase with the complexity of organisms.^2-4^ Introns-early theory holds that introns already existed in ancient ancestor prokaryotes and intron loss allowed the current organisms to have intronless or intron-poor genomes.^5-7^ This theory is supported by the facts that the ancestral eukaryotic forms contained intron-rich genomes^8-10^ and the evolution of eukaryotic genes primarily involves intron loss with only a few episodes of intron gain.^11-14^

In recent years, more and more data have been obtained to favor the introns-early theory.^8-13^ However, the proponents of introns-late theory have still not been persuaded. The main reasons, as we understand, come from 2 aspects. One is that no evidence has been obtained to prove the existence of introns in ancestral bacterial protein-coding genes. The other is that introns-early theory cannot explain why intron number and length increase with the complexity of eukaryotic organisms. It must be confessed that addressing these 2 concerns is confronted with great difficulty. First, all ancestral bacteria are not available today. Thus, no ancient bacterial gene samples are available for examination about intron existence. Yet, this problem could be circumvented to some extent by searching and examining horizontally transferred bacterial genes harbored in eukaryotes. Using this approach, we have found an intron-containing bacterial gene harbored in sea anemone, which suggests possible existence of introns in ancestral bacterial genes.^15^ Second, the lengths and base sequences of introns vary greatly across various organisms. With a group of introns having different lengths, currently there is no measure to find out whether they are evolved from a longer ancestral sequence through gradual reduction in length or from a shorter ancestral sequence through gradual increase in length. Phylogenetic analysis has been widely used to infer evolutionary patterns of gene and protein sequences. However, it is inefficient for studying the evolutionary pattern of introns because the phylogenetic tree formed by intron sequences generally has very poor statistical support, based on which no evolutionary pattern can be inferred to explain how intron sequences have evolved.

Sequence simulation is an important measure for evolutionary studies. A considerable number of statistical models and methods have been developed and tested for inferring the evolutionary relationship of nucleotide and protein sequences.^16-18^ The established models are effective in simulating evolution of real sequences,^19,20^ in establishing databases of simulated protein alignments,^21^ and in exploring early events in the ecological differentiation of bacterial genomes.^22^ However, these methods were mostly developed for simulation of gene or protein sequences which possess high conservatism. They are not suitable for simulation of intron sequences that have undergone high number of nucleotide mutation and large pieces of nucleotide deletion or insertion. Therefore, in this study, we designed and constructed 2 types of evolutionary models, that is, mutation-and-deletion (MD) and mutation-and-insertion (MI), to simulate the evolution of introns from a chordate gene using randomly generated and mutated sequences. Then, we compared attributes of model-generated sequences with real chordate introns. The results show that the MD model with proper parameter settings could generate sequences that have attributes matchable to chordate introns, whereas the MI model with any parameter settings failed in doing so. These results suggest that the surveyed chordate introns should have evolved from a longer ancestral sequence through gradual reduction in length. The established methodology provides an effective measure to study the evolutionary pattern of introns from organisms of specific taxonomic groups.

## Materials and Methods

### Chordate introns and their attributes

**Figure 1.**
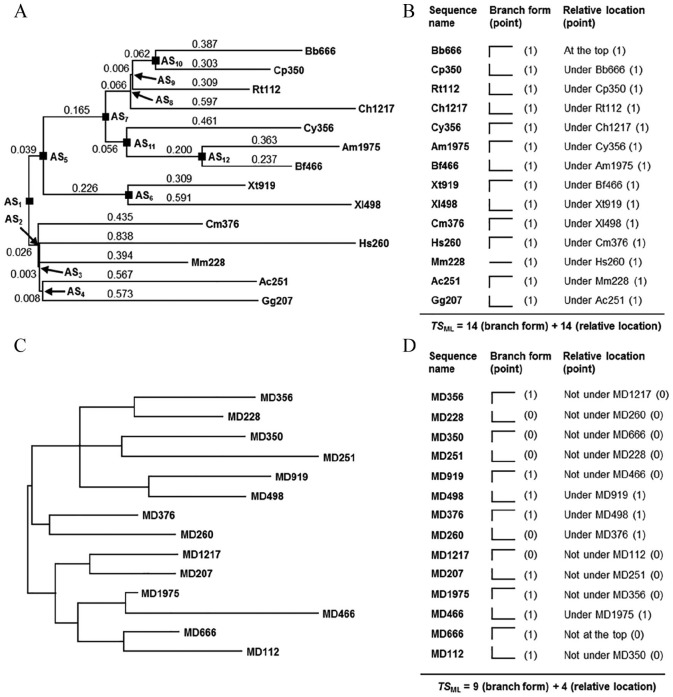
Phylogenetic trees and *TS*_ML_ value calculation of nucleotide sequences. (A) Original maximum likelihood (ML) tree constructed using 14 chordate introns. Branch lengths are indicated by values above or below each branch. The 14 chordate introns are given in 2-letter abbreviation of a specific species plus its intron length. Ac: *Aquila chrysaetos* (golden eagle), Am: *Astyanax mexicanus* (Mexican tetra), Bb: *Branchiostoma belcheri* (Belcher’s lancelet), Bf: *Branchiostoma floridae* (Florida lancelet), Ch: *Clupea harengus* (Atlantic herring), Cm: *Callorhinchus milii* (elephant shark), Cp: *Crocodylus porosus* (Australian saltwater crocodile), Cy: *Chelonia mydas* (green sea turtle), Gg: *Gallus gallus* (chicken), Hs: *Homo sapiens* (human), Mm: *Mus musculus* (house mouse), Rt: *Rhincodon typus* (whale shark), Xl: *Xenopus laevis* (African clawed frog), and Xt: *Xenopus tropicalis* (tropical clawed frog). (B) *TS*_ML_ value calculation of 14 chordate introns. There are 3 branch forms, namely, L-shape, inverted L-shape, and straight line. Relative location of an intron is simply indicated by telling it is under which other intron. Each chordate intron is given 1 point for its branch form and relative location, respectively. Thus, the tree shown in panel (A) has a *TS*_ML_ value of 28. (C) Original ML tree constructed using 14 sequences generated by an MD (mutation-and-deletion) model. For simplicity, branch lengths are not shown in the tree. (D) *TS*_ML_ value calculation of 14 MD model–generated sequences. The branch form and relative location of each model-generated sequence are compared with those of the chordate intron with the same length. If a model-generated sequence has the same branch form or relative location in the tree as its correspondent chordate intron, it is given 1 point. Otherwise, it is given 0 point. So, the tree shown in panel (C) has a *TS*_ML_ value of 13. AS indicates ancestral sequence.

The gene segment coding for bHLH (basic helix-loop-helix) motif of *TF4* (transcription factor–like protein 4) has only 1 phase zero intron in chordates. Exon sequences flanking this intron are highly conserved. This intron was selected as the research subject to ensure that the introns of different chordates come from the common ancestor. This intron is 112 to 1975 bases long in the 14 species chosen to represent various classes of chordates (Table 1). These 14 intron sequences were aligned using Muscle program^23^ first and then loaded into MEGA 5.2 software^24^ to generate the original phylogenetic tree (Figure 1A) using maximum likelihood (ML) algorithm. Afterward, MEGA 5.2 and the constructed ML tree were used to determine 5 attributes of these chordate introns, namely, *L*_MSA_ (length of multiple sequence alignment), *R*_K2+I_ (ratio of transition to transversion under K_2+I_ parameter model^25^), $\bar{D}$ (overall mean distance), $SE_{\bar{D}}$ (standard error of the overall mean distance), and *TS*_ML_ (topology score of the constructed ML tree), which were found to be 2139 bases, 1.53, 1.084, 0.089, and 28, respectively. Detailed steps for determining these attributes are described in Supplementary File 1.

**Table 1.** Fourteen species selected to represent various classes of chordates.

Class	Order	Family	Species
Not available	Not available	Branchiostomidae	*Branchiostoma floridae* (Florida lancelet)
			*Branchiostoma belcheri* (Belcher’s lancelet)
Chondrichthyes	Orectolobiformes	Rhincodontidae	*Rhincodon typus* (whale shark)
	Chimaeriformes	Callorhinchidae	*Callorhinchus milii* (elephant shark)
Actinopteri	Clupeiformes	Clupeidae	*Clupea harengus* (Atlantic herring)
	Characiformes	Characidae	*Astyanax mexicanus* (Mexican tetra)
Amphibia	Anura	Pipidae	*Xenopus laevis* (African clawed frog)
			*Xenopus tropicalis* (tropical clawed frog)
Sauropsida	Crocodylia	Crocodylidae	*Crocodylus porosus* (Australian saltwater crocodile)
	Testudines	Cheloniidae	*Chelonia mydas* (green sea turtle)
Aves	Galliformes	Phasianidae	*Gallus gallus* (chicken)
	Falconiformes	Accipitridae	*Aquila chrysaetos* (golden eagle)
Mammalia	Rodentia	Muridae	*Mus musculus* (house mouse)
	Primates	Hominidae	*Homo sapiens* (human)

### Simulation of intron evolution

To simulate intron evolution, we assume that the 14 chordate introns are evolved from 1 common ancestral sequence (AS_1_) through gradual reduction/increase in length accompanied by base mutation (Figure 1A). The AS_1_ sequence was generated using a C++ program, in which 4 integers (1, 2, 3, and 4) were generated at random and were referred to bases A, G, T, and C, respectively. After a sequence of demanded length was obtained, its first and last 2 bases were replaced by GT and AG to mimic the structure of an intron. Starting from the AS_1_ sequence, the formation of each chordate intron can be divided into separate stages. For instance, intron Cm376 (an intron of 376 bp from the elephant shark, *Callorhinchus milii*) was evolved from AS_2_, and AS_2_ itself was evolved from AS_1_. The evolutionary models we constructed have 5 adjustable parameters, that is, *L*_AS1_ (length of ancestral sequence 1), *L*_AS12_ (length of ancestral sequence 12), *M*_1_ (mutated bases per 1 branch length), *L*_I/D_ (length of bases inserted or deleted each time), and *M*_I/D_ (number of bases mutated each time). Parameters *L*_AS1_, *L*_AS12_, and *M*_1_ define how many bases should be deleted/inserted and mutated at each stage, whereas *L*_I/D_ and *M*_I/D_ define how many bases should be deleted/inserted and mutated each time within a stage. Once the values of these 5 parameters are set, an evolutionary event for a specific intron sequence can be simulated. As an example, Supplementary File 2 describes detailed steps of how to generate a 376-base sequence for simulating evolution of intron Cm376 using the MD model. The evolution of all other ancestral sequences and intron sequences can be simulated in the similar way.

### Determination of L_AS12_ length

As shown in Figure 1A, there are 12 ancestral sequences for the 14 chordate introns. Among them, AS_2_ to AS_4_, AS_6_, and AS_8_ to AS_12_ are directly ancestral to certain chordate introns. Because our evolutionary models assume consecutive deletion or insertion during evolution, the last ancestral sequence (*L*_AS12_) should have a valid length to ensure simulation of intron evolution. That is to say, in the MD model, AS_2_ to AS_4_, AS_6_, and AS_8_ to AS_12_ must be longer than the correspondent introns evolved from them. As such, the minimum value of *L*_AS12_ was set to 2000 bases (Table 2) to ensure validity of using it to simulate the formation of intron Am1975 (the longest among 14 chordate introns). And in the MI model, AS_2_ to AS_4_, AS_6_, and AS_8_ to AS_12_ must be shorter than the correspondent introns evolved from them. Thus, the maximum value of *L*_AS12_ was set to 140 bases (Table 2) to ensure that AS_9_ is shorter than Rt112 (the shortest among 14 chordate introns).

**Table 2.** Factor and level design for evolution models using L_16_(4*5) orthogonal table.

Evolution model	Level	Factors				
		*L*_AS1_	*L*_AS12_	*M*_1_	*L*_I/D_	*M*_I/D_
Mutation-and-deletion	1	4000	2000	200	31-50	11-20
	2	5000	2250	400	71-90	21-30
	3	6000	2500	600	111-130	31-40
	4	7000	2750	800	151-170	41-50
Mutation-and-insertion	1	10	110	200	31-50	11-20
	2	20	120	400	71-90	21-30
	3	30	130	600	111-130	31-40
	4	40	140	800	151-170	41-50

Abbreviations: *L*_AS1_, length of ancestral sequence 1; *L*_AS12_, length of ancestral sequence 12; *L*_I/D_, length of bases inserted or deleted each time; *M*_1_, mutated bases per 1 branch length; *M*_I/D_, number of bases mutated each time.

### R value of constructed models

The transition to transversion ratio (*R*) is adjustable in MD and MI models. However, a fixed value is given to it prior to model running in accordance with the attributes of intron sequences for study. This value is set to 1.5 in both MD and MI models because the *R* value of the 14 chordate introns is 1.53 as revealed by model testing (see the first paragraph of “Materials and Methods” section).

### Statistical analysis

Statistical analyses were performed using SPSS software (version 17.0). For data from orthogonally designed models, “univariate” analysis was conducted to evaluate the effects of various factors on attributes of model-generated sequences. Significance of variance was analyzed through comparing the main effect of each factor without investigating the interaction between factors. Duncan post hoc test was used to conduct multiple comparisons for observed means, which were then plotted to view the optimal level of each factor. For data from optimized MD and MI models, “independent samples *t* test” was conducted to compare the difference of attributes between chordate introns and model-generated sequences.

## Results

### Design and construction of evolutionary models

The ML tree obtained from previous step (Figure 1A) was referenced for the design and construction of evolutionary models. In this tree, the first ancestral sequence (AS_1_) was evolved to form AS_2_ to AS_12_, which were then evolved to form the 14 chordate introns. The formation of each ancestral sequence or chordate intron sequence was considered as the result of both base mutation and base deletion/insertion, which are related to evolutionary distance shown above or below each branch of the tree. In other words, each sequence in the tree (except AS_1_) was evolved from its specific AS after a target number of bases was mutated and a target length of bases was deleted or inserted. Nevertheless, base mutation and base deletion/insertion are a continuous process, meaning that a target number of bases to be mutated and a target length of base deletion/insertion should be completed at consecutive stages. Therefore, we have designed and constructed evolutionary models to simulate base mutation and base deletion/insertion alternately before the target number of bases is mutated and the target length of bases is deleted/inserted. In addition, the evolutionary models were designed to receive parameters in batch so that a set of 14 sequences could be generated at one time. Detailed flow charts for the construction of MD and MI models are shown in Figures S1 and S2, respectively. The constructed MD model assumes that the 14 chordate introns were evolved from a fairly long AS_1_ sequence through a gradual reduction in length accompanied by base mutation, whereas the constructed MI model assumes that the 14 chordate introns were evolved from a fairly short AS_1_ sequence through a gradual increase in length accompanied by base mutation. Both programs were compiled with C++ language.

### Orthogonal tests of MD and MI models

The MD or MI model we constructed has 5 adjustable parameters. To understand the influence of different values of these parameters on attributes of the 14 model-generated sequences, we have designed tests using L_16_(4*5) orthogonal table (Table 2). The 5 adjustable parameters are *L*_AS1_, *L*_AS12_, *M*_1_, *L*_I/D_, and *M*_I/D_. Among them, *L*_I/D_ and *M*_I/D_ are not given in fixed values because base deletion/insertion and base mutation are believed not to occur in fixed numbers. After assigning 4 levels to these 5 parameters/factors, 16 orthogonal tests were designed and run for MD and MI models, respectively. Each test generated 10 sets of sequences for determining their attributes. The obtained results are listed in Tables S1 and S2.

**Figure 2.**
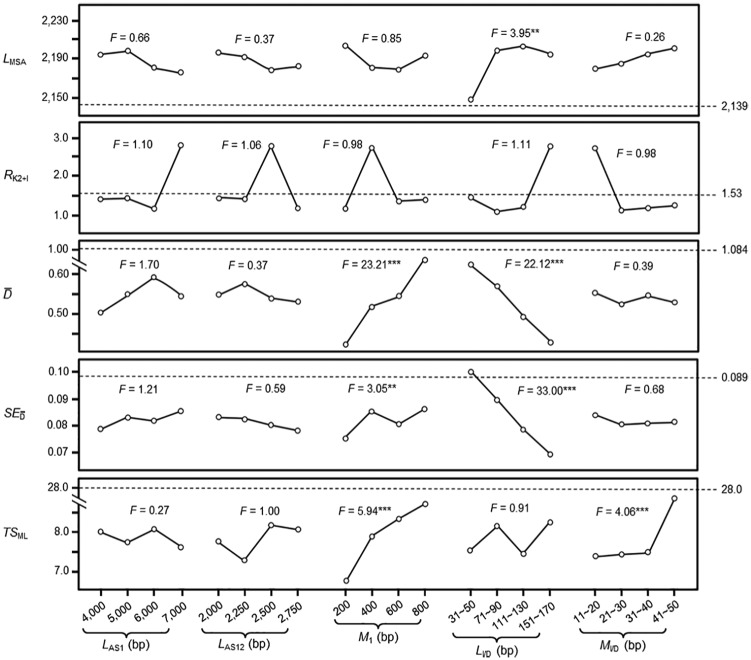
Effects of different factors on testing result of mutation-and-deletion model. Different factors at various levels are given in the horizontal axis. The 5 factors are *L*_AS1_ (length of ancestral sequence 1), *L*_AS12_ (length of ancestral sequence 12), *M*_1_ (mutated bases per 1 branch length), *L*_I/D_ (length of bases inserted or deleted each time), and *M*_I/D_ (number of bases mutated each time). Attributes of model-generated sequences are given in the vertical axis. They are shown as *L*_MSA_ (length of multiple sequence alignment), *R*_K2+I_ (ratio of transition to transversion under K_2+I_ parameter model), $\bar{D}$ (overall mean distance), $SE_{\bar{D}}$ (standard error of the overall mean distance), and *TS*_ML_ (topology score of the constructed ML tree). *F* value is from analysis of variance through comparing the main effect of each factor without investigating the interaction between factors. Dashed lines mark the attributes of chordate intron sequences. *, **, and *** indicate significant effect of various factors on attributes of model-generated sequences at *P* < .1, *P* < .05, and *P* < .01 level, respectively.

**Figure 3.**
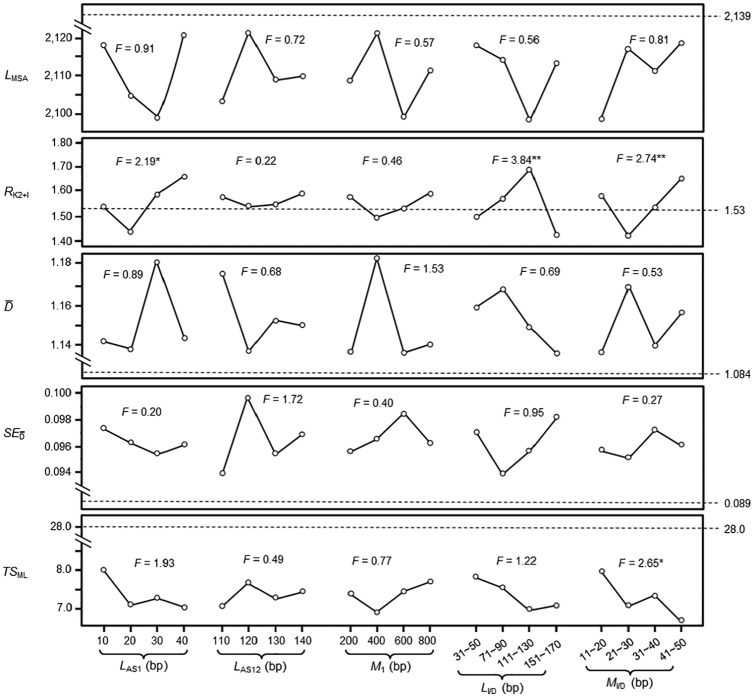
Effects of different factors on testing result of mutation-and-insertion model. Different factors at various levels are given in the horizontal axis. The 5 factors are *L*_AS1_ (length of ancestral sequence 1), *L*_AS12_ (length of ancestral sequence 12), *M*_1_ (mutated bases per 1 branch length), *L*_I/D_ (length of bases inserted or deleted each time), and *M*_I/D_ (number of bases mutated each time). Attributes of model-generated sequences are given in the vertical axis. They are shown as *L*_MSA_ (length of multiple sequence alignment), *R*_K2+I_ (ratio of transition to transversion under K_2+I_ parameter model), $\bar{D}$ (overall mean distance), $SE_{\bar{D}}$ (standard error of the overall mean distance), and *TS*_ML_ (topology score of the constructed ML tree). *F* value is from analysis of variance through comparing the main effect of each factor without investigating the interaction between factors. Dashed lines mark the attributes of chordate intron sequences. * and ** indicate significant effect of various factors on attributes of model-generated sequences at *P* < .1 and *P* < .05 level, respectively.

Statistical analysis to these data indicated that, in sequences generated by the MD model, *L*_MSA_ is affected significantly by *L*_I/D_; $\bar{D}$ and $SE_{\bar{D}}$ are affected significantly by *M*_1_ and *L*_I/D_; *TS*_ML_ is affected significantly by *M*_1_ and *M*_I/D_; and *R*_K2+I_ is not significantly affected by any parameters (Figure 2). In sequences generated by the MI model, *R*_K2+I_ is affected significantly by *L*_AS1_, *L*_I/D_, and *M*_I/D_; *TS*_ML_ is affected significantly by *M*_I/D_; and *L*_MSA_, $\bar{D}$, and $SE_{\bar{D}}$ are not significantly affected by any parameters (Figure 3).

### Parameter optimization for running the MD model

In Figure 2, the attributes of 14 chordate introns are indicated at the right margin. These are the target values for optimizing model parameters. By referencing to Figure 2, our first test on parameter optimization for running the MD model (MD_17_) uses 6000 bases as *L*_AS1_, 2000 bases as *L*_AS12_, 600 bases as *M*_1_, 31 to 50 bases as *L*_I/D_, and 21 to 30 bases as *M*_I/D_ (Table 3). Among the 5 attributes of sequences generated by MD_17_, $\bar{D}$ value is significantly lower than that of the 14 chordate introns at *P* < .05 level. Here, it is to be noted that the attributes of 14 chordate introns are also presented as “M ± SD” (n = 10), which are obtained from allowing each of the 14 chordate intron sequences to mutate by only 1 base (Table 3). This was an adjustment after we became aware of the inadequacy in using 28 points as the target value for *TS*_ML_. As we have found, after each of the 14 chordate intron sequences is allowed to mutate by only 1 base, the resultant *TS*_ML_ will generally drop to below 16. So, we repeated such 1-base mutation to the 14 chordate introns (using another C++ program compiled by us) for 10 times and thus obtained their attributes in the “M ± SD” form.

**Table 3.** Attributes of sequences generated from optimized MD and MI models.

Test no.	Model parameters					Attributes of generated sequences				
	*L*_AS1_	*L*_AS12_	*M*_1_	*L*_I/D_	*M*_I/D_	*L*_MSA_	*R*_K2+I_	$\bar{D}$	$SE_{\bar{D}}$	*TS*_ML_
FCIs	/	/	/	/	/	2144 ± 62	1.41 ± 0.24	0.93 ± 0.13	0.101 ± 0.010	10.0 ± 3.6
MD_17_	6000	2000	600	31-50	21-30	2158 ± 63	1.52 ± 0.37	0.79 ± 0.12**	0.098 ± 0.006	9.0 ± 2.4
MD_18_	6000	2500	800	31-50	21-30	2140 ± 60	1.50 ± 0.38	0.81 ± 0.13*	0.095 ± 0.009	8.8 ± 1.6
MD_19_	6000	2000	800	31-50	41-50	2129 ± 67	1.54 ± 0.58	0.83 ± 0.12*	0.107 ± 0.011	9.9 ± 2.8
MD_20_	5000	2500	800	31-50	21-30	2152 ± 75	1.65 ± 0.35*	0.82 ± 0.23	0.095 ± 0.009	7.4 ± 2.5*
MD_21_	6000	2250	800	31-50	11-20	2158 ± 87	1.50 ± 0.28	0.83 ± 0.09*	0.099 ± 0.014	9.7 ± 2.0
MD_22_	6000	2250	800	31-50	41-50	2154 ± 83	1.67 ± 0.32*	0.89 ± 0.14	0.106 ± 0.009	8.7 ± 2.9
MD_23_	6000	2000	800	31-50	21-30	2187 ± 87	1.53 ± 0.22	0.88 ± 0.11	0.098 ± 0.009	8.2 ± 2.4
MD_24_	6000	2500	800	31-50	11-20	2143 ± 44	1.41 ± 0.26	0.88 ± 0.11	0.099 ± 0.008	8.4 ± 1.7
MI_17_	10	120	200	31-50	31-40	2097 ± 48*	1.49 ± 0.37	1.10 ± 0.11***	0.101 ± 0.010	7.0 ± 1.9**
MI_18_	10	120	200	31-50	11-20	2102 ± 83	1.50 ± 0.33	1.03 ± 0.12*	0.104 ± 0.006	6.0 ± 2.2***
MI_19_	10	120	800	31-50	11-20	2123 ± 84	1.35 ± 0.19	1.14 ± 0.11***	0.094 ± 0.008*	8.4 ± 2.3
MI_20_	20	120	200	31-50	11-20	2123 ± 57	1.29 ± 0.43	1.26 ± 0.17***	0.097 ± 0.012	7.5 ± 2.0*
MI_21_	10	110	800	31-50	11-20	2105 ± 70	1.53 ± 0.23	1.22 ± 0.11***	0.088 ± 0.006***	7.5 ± 2.1*
MI_22_	40	110	200	31-50	11-20	2082 ± 62**	1.55 ± 0.30	1.21 ± 0.09***	0.098 ± 0.008	8.2 ± 1.9
MI_23_	20	120	200	151-170	11-20	2138 ± 43	1.34 ± 0.26	1.11 ± 0.13***	0.098 ± 0.012	6.9 ± 1.6**
MI_24_	10	120	800	151-170	11-20	2143 ± 68	1.47 ± 0.43	1.13 ± 0.22**	0.091 ± 0.013*	7.0 ± 3.2*

Abbreviations: FCIs, 14 chordate introns. *L*_AS1_, length of ancestral sequence 1, *L*_AS12_, length of ancestral sequence 12; *L*_I/D_, length of bases inserted or deleted each time; *L*_MSA_, length of multiple sequence alignment; *M*_1_, mutated bases per 1 branch length; MD, mutation-and-deletion; MI, mutation-and-insertion; *M*_I/D_, number of bases mutated each time; *R*_K2+I_, ratio of transition to transversion under K_2+I_ parameter model; $\bar{D}$: overall mean distance; $SE_{\bar{D}}$, standard error of the overall mean distance; *TS*_ML_, topology score of the constructed ML tree.

Attributes of FCIs are obtained from allowing each of the sequence to mutate by only 1 base. Data are presented as M ± SD (n = 10).

*, **, and *** indicate significant difference from independent *t* test compared with FCIs at *P* < .1, *P* < .05, and *P* < .01 level, respectively.

From Figure 2, we can see that $\bar{D}$ value is significantly affected by *M*_1_ and *L*_I/D_. So, in test MD_18_, we used 2500 bases as *L*_AS12_ and 800 bases as *M*_1_ while keeping other parameters unchanged. But sequences generated by MD_18_ still have significant difference in $\bar{D}$ value, though at the *P* < .1 level. After this, we have tried various values for parameters *L*_AS1_, *L*_AS12_, and *M*_I/D_ to run the MD model (tests MD_19_-MD_24_). It was found that 1 or 2 of the 5 attributes of sequences generated by tests MD_19_ to MD_22_ are still significantly different from those of the 14 chordate introns. However, all 5 attributes of sequences generated in tests MD_23_ and MD_24_ have no significant difference with chordate introns (Table 3). Thus, it is concluded that the MD model with proper parameter setting can generate sequences with attributes matchable to chordate introns.

### Parameter optimization for running the MI model

By referencing to Figure 3, our first test on parameter optimization for running the MI model (MI_17_) uses 10 bases as *L*_AS1_, 120 bases as *L*_AS12_, 200 bases as *M*_1_, 31 to 50 bases as *L*_I/D_, and 31 to 40 bases as *M*_I/D_ (Table 3). However, 3 attributes (*L*_MSA_, $\bar{D}$, and *TS*_ML_) of the model-generated sequences are significantly different from those of chordate introns. So, in test MI_18_, we changed *M*_I/D_ to 11 to 20 bases while keeping other parameters unchanged. The model-generated sequences have no significant difference in *L*_MSA_ but have significantly higher $\bar{D}$ value and significantly lower *TS*_ML_ value. After this, we have tried various values for all 5 parameters to run the MI model (tests MI_19_-MI_24_). It was found that 2 or 3 of the 5 attributes of sequences generated by tests MI_19_-MI_24_ are always significantly different from those of the 14 chordate introns (Table 3). Thus, it is concluded that the MI model could not generate sequences with all attributes matchable to chordate introns.

## Discussion

Introns are well known for their high variation not only in length but also in base sequence. So far, no common sequence structures/features have been found in introns except those containing transposable elements^26,27^ and microRNAs.^28^ The unavailability of common sequence features makes it very difficult to study the evolutionary pattern of introns through phylogenetic analysis because intron sequences generally yield a phylogenetic tree with very poor statistical support. This is true even when the introns are from the same location of a gene in closely related organisms. For example, the bootstrap values in ML tree shown in Figure 1A are ranging from 3 to 56 (for simplicity, bootstrap values are not shown in the figure). With such low bootstrap support, no evolutionary pattern can be inferred for these intron sequences. As such, it is not clear whether these introns have evolved from a longer ancestral sequence or from a shorter ancestral sequence. In this study, we designed 2 evolutionary models to simulate the evolution of chordate introns. The obtained data (Table 3) demonstrate that the 14 chordate introns should have evolved from a longer ancestral sequence through gradual reduction in length accompanied by base mutation, that is, in a mutation-and-deletion pattern. According to our simulation, the 14 chordate introns probably had a common ancestral sequence of 6000 bp. This ancestral sequence could have undergone 11 to 30 bases mutation and 31 to 50 bases deletion alternately to yield the intron sequences currently existing in various chordate species, and a transition to transversion ratio of 1.5 occurred in base mutation. Although the above simulation is highly dependent on parameter settings, it does provide an effective measure for inferring the evolutionary pattern of intron sequences. At least, it can tell us whether an MD or an MI model better describes the evolutionary pattern of surveyed introns.

So far, studies about evolution of intron sequences have mainly focused on the presence or absence of introns in certain genes across various organisms. Through investigating a large number of intron-gain and intron-loss events, it has been revealed that the evolution of eukaryotic genes primarily involves intron loss.^11-14^ While previous studies have made it clear that intron number has been reduced, it remains unclear whether intron length has been increased or decreased during intron evolution. Our present work provides an example of intron length reduction during the evolution of chordate *TF4* gene. Theoretically, the established methodology can be used to study the evolution of introns at different numbers and lengths and from different organisms because the intron sequences for study are only used to construct the original ML tree (eg, Figure 1A), which is then used as a roadmap to set parameter values accordingly in testing the constructed evolutionary models. Prior to this study, the same models had been applied to simulate the evolution of 11 insect introns with lengths ranging from 299 to 3026 bp. The statistical result showed that the MD model could generate sequences with attributes matchable to insect introns (unpublished data). Our simulations to evolution of introns from other taxonomic groups including fishes, birds, and invertebrates are ongoing. Primary data obtained so far show that the MD model is more likely the pattern followed by intron evolution in these organisms. It is anticipated that more examples of intron length reduction will be found using this approach, showing that introns have been reduced not only in number but also in length during evolution.

Then, if intron number and length have been substantially reduced during evolution, why do intron number and length increase with the complexity of eukaryotic organisms? This is the main supportive fact of introns-late theory that has not been given a rational explanation from introns-early theory. Here, we propose our explanation to this question: higher organisms are not as efficient as lower organisms in reducing number and length of introns. This explanation is consistent with frequent intron loss in yeast,^29^ higher intron retention rate in human than in fruit fly and nematode,^30^ reductive evolution of genomes in complex archaeal ancestor,^31^ and intron-rich ancestor of eukaryotes.^32^ This higher efficiency in lower organisms could be due to higher reproduction rates of lower organisms than higher organisms because frequent genome replication provides more chances for genome streamlining.^33,34^ Therefore, more and longer introns existing in higher organisms are not the result of continuous intron gain, but are the result of low efficiency in reducing intron number and length. This may be considered new evidence to support the introns-early theory and to persuade the proponents of introns-late theory.

## Conclusions

In this study, through designing and constructing MD and MI evolutionary models to simulate the evolution of 14 chordate introns, we found that these chordate introns should have evolved from a longer sequence through gradual reduction in length accompanied by random base mutation. Although successful simulation seems to be highly dependent on parameter settings for the constructed models, it does provide an effective measure to infer the evolutionary pattern of introns, especially in view of intron length variation. The established methodology is expected to facilitate more studies on evolutionary pattern of intron sequences from organisms of various taxonomic groups.

## Supplemental Material

Supplementary_file_1_xyz299583cdc1c74 – Supplemental material for Simulation of Chordate Intron Evolution Using Randomly Generated and Mutated Base Sequences Click here for additional data file. Supplemental material, Supplementary_file_1_xyz299583cdc1c74 for Simulation of Chordate Intron Evolution Using Randomly Generated and Mutated Base Sequences by Guang-Dong Wang, Yong Wang, Zhen Zeng, Jun-Ming Mao, Qin-Liu He, Qin Yao and Ke-Ping Chen in Evolutionary Bioinformatics

Supplementary_file_2_xyz299586f3629a1 – Supplemental material for Simulation of Chordate Intron Evolution Using Randomly Generated and Mutated Base Sequences Click here for additional data file. Supplemental material, Supplementary_file_2_xyz299586f3629a1 for Simulation of Chordate Intron Evolution Using Randomly Generated and Mutated Base Sequences by Guang-Dong Wang, Yong Wang, Zhen Zeng, Jun-Ming Mao, Qin-Liu He, Qin Yao and Ke-Ping Chen in Evolutionary Bioinformatics

Table_S1_xyz29958520c37c8 – Supplemental material for Simulation of Chordate Intron Evolution Using Randomly Generated and Mutated Base Sequences Click here for additional data file. Supplemental material, Table_S1_xyz29958520c37c8 for Simulation of Chordate Intron Evolution Using Randomly Generated and Mutated Base Sequences by Guang-Dong Wang, Yong Wang, Zhen Zeng, Jun-Ming Mao, Qin-Liu He, Qin Yao and Ke-Ping Chen in Evolutionary Bioinformatics

Table_S2_xyz299588fc8496b – Supplemental material for Simulation of Chordate Intron Evolution Using Randomly Generated and Mutated Base Sequences Click here for additional data file. Supplemental material, Table_S2_xyz299588fc8496b for Simulation of Chordate Intron Evolution Using Randomly Generated and Mutated Base Sequences by Guang-Dong Wang, Yong Wang, Zhen Zeng, Jun-Ming Mao, Qin-Liu He, Qin Yao and Ke-Ping Chen in Evolutionary Bioinformatics

## References

[1] Doolittle WF Stoltzfus A Molecular evolution: genes-in-pieces revisited. Nature. 1993;361:403. 8429878 10.1038/361403a0

[2] Logsdon JM Jr The recent origins of spliceosomal introns revisited. Curr Opin Genet Dev. 1998;8:637-648. 9914210 10.1016/s0959-437x(98)80031-2

[3] Penny D Hoeppner MP Poole AM Jeffares DC An overview of the introns-first theory. J Mol Evol. 2009;69:527-540. 19777149 10.1007/s00239-009-9279-5

[4] Rodríguez-Trelles F Tarrío R Ayala FJ Origins and evolution of spliceosomal introns. Annu Rev Genet. 2006;40:47-76. 17094737 10.1146/annurev.genet.40.110405.090625

[5] Doolittle WF Genes in pieces: were they ever together? Nature. 1978;272: 581-582.

[6] Gilbert W The exon theory of genes. Cold Spring Harb Symp Quant Biol. 1980;52:901-905. 10.1101/sqb.1987.052.01.098 2456887

[7] Roy SW Recent evidence for the exon theory of genes. Genetica. 2003;118:251-266. 12868614

[8] Csuros M Likely scenarios of intron evolution. In: McLysaght A Huson DH, eds. Comparative genomics (Lecture Notes in Computer Science), vol. 3678 Berlin: Springer; 2005:47-60.

[9] Niu DK Hou WR Li SW mRNA-mediated intron losses: evidence from extraordinarily large exons. Mol Biol Evol. 2005;22:1475-1481. 15788745 10.1093/molbev/msi138

[10] Rogozin IB Carmel L Csuros M Koonin EV Origin and evolution of spliceosomal introns. Biol Direct. 2012;7:11. doi:10.1186/1745-6150-7-11. 22507701 PMC3488318

[11] Carmel L Wolf YI Rogozin IB Koonin EV Three distinct modes of intron dynamics in the evolution of eukaryotes. Genome Res. 2007;17:1034-1044. 17495008 10.1101/gr.6438607 PMC1899114

[12] Csuros M Rogozin IB Koonin EV A detailed history of intron-rich eukaryotic ancestors inferred from a global survey of 100 complete genomes. PLoS Comput Biol. 2011;7:e1002150. 10.1371/journal.pcbi.1002150 PMC3174169 21935348

[13] Yang YF Zhu T Niu DK Association of intron loss with high mutation rate in Arabidopsis: implications for genome size evolution. Genome Biol Evol. 2013;5:723-733. 23516254 10.1093/gbe/evt043 PMC4104619

[14] Roy SW Is genome complexity a consequence of inefficient selection? Evidence from intron creation in nonrecombining regions. Mol Biol Evol. 2016;33:3088-3094. 27655009 10.1093/molbev/msw172

[15] Wang Y Tao XF Su ZX, et al Current bacterial gene encoding capsule biosynthesis protein CapI contains nucleotides derived from exonization. Evol Bioinform Online. 2016;12:303-312. 27980385 10.4137/EBO.S40703 PMC5154736

[16] Ashkenazy H Levy Karin E Mertens Z Cartwright RA Pupko T SpartaABC: a web server to simulate sequences with indel parameters inferred using an approximate Bayesian computation algorithm. Nucleic Acids Res. 2017;45:W453-W457. doi:10.1093/nar/gkx322. 28460062 PMC5570005

[17] Peng B Chen HS Mechanic LE, et al Genetic data simulators and their applications: an overview. Genet Epidemiol. 2015;39:2-10. 25504286 10.1002/gepi.21876 PMC4804465

[18] Stoye J Evers D Meyer F Rose: generating sequence families. Bioinformatics. 1998;14:157-163. 9545448 10.1093/bioinformatics/14.2.157

[19] Goldman N Simple diagnostic statistical tests of models for DNA substitution. J Mol Evol. 1993;37:650-661. 8114118 10.1007/BF00182751

[20] Goldman N Statistical tests of models of DNA substitution. J Mol Evol. 1993;36:182-198. 7679448 10.1007/BF00166252

[21] Pervez MT Shah HA Babar ME Naveed N Shoaib M SAliBASE: a database of simulated protein alignments. Evol Bioinform Online. 2019;15:1176934318821080. doi:10.1177/1176934318821080. PMC6343434 30733625

[22] Shapiro BJ Friedman J Cordero OX, et al Population genomics of early events in the ecological differentiation of bacteria. Science. 2012;336:48-51. 22491847 10.1126/science.1218198 PMC3337212

[23] Edgar RC MUSCLE: multiple sequence alignment with high accuracy and high throughput. Nucleic Acids Res. 2004;32:1792-1797. 15034147 10.1093/nar/gkh340 PMC390337

[24] Tamura K Peterson D Peterson N Stecher G Nei M Kumar S MEGA5: molecular evolutionary genetics analysis using maximum likelihood, evolutionary distance, and maximum parsimony methods. Mol Biol Evol. 2011;28:2731-2739. 21546353 10.1093/molbev/msr121 PMC3203626

[25] Kimura M A simple method for estimating evolutionary rate of base substitution through comparative studies of nucleotide sequences. J Mol Evol. 1980;16:111-120. 7463489 10.1007/BF01731581

[26] Nene V Wortman JR Lawson D, et al Genome sequence of *Aedes aegypti*, a major arbovirus vector. Science. 2007;316:1718-1723. 17510324 10.1126/science.1138878 PMC2868357

[27] Zhang DB Wang Y Liu AK, et al Phylogenetic analyses of vector mosquito basic helix-loop-helix transcription factors. Insect Mol Biol. 2013;22:608-621. 23906262 10.1111/imb.12049

[28] Chorev M Carmel L Computational identification of functional introns: high positional conservation of introns that harbor RNA genes. Nucleic Acids Res. 2013;41:5604-5613. 23605046 10.1093/nar/gkt244 PMC3675471

[29] Yin LF Hu MJ Wang F, et al Frequent gain and loss of introns in fungal cytochrome b genes. PLoS ONE. 2012;7:e49096. doi:10.1371/journal.pone.0049096. PMC3492308 23145081

[30] Bányai L Patthy L Evidence that human genes of modular proteins have retained significantly more ancestral introns than their fly or worm orthologues. FEBS Lett. 2004;565:127-132. 15135065 10.1016/j.febslet.2004.03.088

[31] Corradi N Slamovits CH The intriguing nature of microsporidian genomes. Brief Funct Genomics. 2011;10:115-124. 21177329 10.1093/bfgp/elq032

[32] Wolf YI Koonin EV Genome reduction as the dominant mode of evolution. BioEssays. 2013;35:829-837. 23801028 10.1002/bies.201300037 PMC3840695

[33] Koonin EV Evolution of genome architecture. Int J Biochem Cell Biol. 2009;41: 298-306. 18929678 10.1016/j.biocel.2008.09.015 PMC3272702

[34] Swan BK Tupper B Sczyrba A, et al Prevalent genome streamlining and latitudinal divergence of planktonic bacteria in the surface ocean. Proc Natl Acad Sci USA. 2013;110:11463-11468. 23801761 10.1073/pnas.1304246110 PMC3710821

