# Supplementary material for: Simulation of Chordate Intron Evolution Using Randomly Generated and Mutated Base Sequences
Source: Evol Bioinform Online. 2020 Jan 29;16:1176934320903108. doi: 10.1177/1176934320903108 (PMC6990610; doi:10.1177/1176934320903108)
Supplement: Supplementary_file_1_xyz299583cdc1c74 – Supplemental material for Simulation of Chordate Intron Evolution Using Randomly Generated and Mutated Base Sequences [file Supplementary_file_1_xyz299583cdc1c74.pdf]

Supplementary file 1. Detailed steps for determining attributes of chordate introns. This file describes detailed steps for determining the five attributes of fourteen chordate introns. Specifically, L_{MSA} was obtained by using Muscle program embedded in MEGA 5.2 software to yield the multiple sequence alignment with default settings. R_{K2+I} was from the result of running "Find Best DNA/Protein Models (ML)" command under "Models" menu of MEGA 5.2, which shows that the best model fitting the fourteen chordate introns is K2+I. Here, "K2" refers to kimura 2 parameter model characterized by equal base frequency and unequal transition to transversion ratio. "I" means that "rates among sites" of the intron sequences have invariant sites. $\bar{D}$ and $SE_{\bar{D}}$ were the result of running "Compute Overall Mean Distance" command under "Distance" menu of MEGA 5.2 with the following parameter settings: variance was estimated using 200 bootstrap replications, K2+I parameter model was chosen as the substitution model and substitutions included both transitions and transversions, rates among sites were set to "Has Invariant Sites", and gaps in the alignment were completely deleted.

The same parameter setting for determining $\bar{D}$ and $SE_{\bar{D}}$ values was used to run "Construct/Test Maximum Likelihood Tree" command under "Phylogeny" menu. Figure 1a shows the constructed original ML tree. In order to evaluate the similarity between two ML trees, we introduced TS_{ML} value to quantify topology of a phylogenetic tree. TS_{ML} is the sum of points each individual sequence is given based on its branch form and relative location in the original ML tree. Please note that, in order to minimize the influence of partial symmetry existing in phylogenetic trees, calculations of TS_{ML} value for all model-generated sequences are based on their original ML trees. Since our present study is aimed to simulate intron evolution from chordate TF4 gene, the original ML tree formed by the fourteen chordate introns is considered as standard tree (Figure 1a). In order to quantify this tree, each intron sequence is given two points for its branch form and relative location. For instances, the intron from *Branchiostoma belcheri* (Bb666) is given one point for its inverted L-shape and one point for its location at the top. The intron from *Crocodylus porosus* (Cp350) is given one point for its L-shape and one point for its location under Bb666. Similarly, each of the rest intron sequences is also given two points for its correspondent branch form and relative location. Thus, this tree has a TS_{ML} value of 28 with 14 points from branch form and relative

location respectively (Figure 1b). Figure 1c shows an original ML tree constructed from one MD (mutation-and-deletion) evolutionary model. After comparing the branch form and relative location with the standard tree in Figure 1a, this tree has a TS_{ML} value of 13 with 9 points from branch form and 4 points from relative location (Figure 1d). Examples of calculation: MD666 (the nucleotide sequence to simulate Bb666 intron) is given one point for its inverted "L" branch form (which is the same with that of Bb666) but is given zero point for its relative location (because it is not at the top). MD350 (the nucleotide sequence to simulate Cp350 intron) is given zero point for its inverted L-shape (which is different with that of Cp350) and zero point for its relative location (which is not under MD666).
