# Supplementary material for: Simulation of Chordate Intron Evolution Using Randomly Generated and Mutated Base Sequences
Source: Evol Bioinform Online. 2020 Jan 29;16:1176934320903108. doi: 10.1177/1176934320903108 (PMC6990610; doi:10.1177/1176934320903108)
Supplement: Supplementary_file_2_xyz299586f3629a1 – Supplemental material for Simulation of Chordate Intron Evolution Using Randomly Generated and Mutated Base Sequences [file Supplementary_file_2_xyz299586f3629a1.pdf]

Supplementary file 2. Detailed steps for simulating evolution of intron Cm376. This file describes detailed steps for simulation of intron Cm376 formation when the five parameters (L_{AS1}, L_{AS12}, M_1, $L_{I/D}$ and $M_{I/D}$) are set to 4,000, 2,000, 200, 31~50 and 11~20 respectively for MD model. The model will generate a 376-base sequence in two steps. First, the 4,000-base AS_1 had 5 bases mutated and 113 bases deleted to form AS_2. Then, AS_2 had 87 bases mutated and 3,511 bases deleted to form the 376-base sequence. Here, the 5-base mutation is calculated using 0.026×200, in which 200 is the number of mutated bases per 1 branch length. The 113-base deletion is calculated using (4,000 – 2,000)*0.026/0.460, in which 4,000 is the length of AS_1, 2,000 is the length of AS_{12}, 0.026 is the branch length from AS_1 to AS_2, and 0.460 is the total branch length from AS_1 to AS_{12} (0.039+0.165+0.056+0.200). The 87-base mutation is calculated using 0.435×200, in which 0.435 is the branch length from AS_2 to Cm376. The 3,511-base deletion is calculated using 3,887 – 376, in which 3,887 is the length of AS_2 and 376 is the length of sequence Cm376 (Figure 1a). Afterwards, to simulate formation of intron Cm376, the 4,000-base AS_1 will have 11~20 bases mutated and 31~50 bases deleted each time until 5 bases are mutated and 113 bases are deleted. Then, the 3,887-base AS_2 will have 11~20 bases mutated and 31~50 bases deleted each time until 87 bases are mutated and 3,511 bases are deleted. Similarly, evolution of all other ancestral sequences and intron sequences can be simulated as described above.
