# Supplementary material for: Simulation of Chordate Intron Evolution Using Randomly Generated and Mutated Base Sequences
Source: Evol Bioinform Online. 2020 Jan 29;16:1176934320903108. doi: 10.1177/1176934320903108 (PMC6990610; doi:10.1177/1176934320903108)
Supplement: Table_S1_xyz29958520c37c8 – Supplemental material for Simulation of Chordate Intron Evolution Using Randomly Generated and Mutated Base Sequences [file Table_S1_xyz29958520c37c8.pdf]

Table S1. Orthogonal test results of MD model

Test	Model parameters					Attributes of generated sequences				
No.	L_{AS1}	L_{AS12}	M_1	$L_{I/D}$	$M_{I/D}$	L_{MSA}	R_{K2+I}	$\bar{D}$	$SE_{\bar{D}}$	TS_{ML}
MD_1	4,000	2,000	200	31~50	11~20	2,175±35	1.46±0.45	0.54±0.22	0.095±0.022	6.1±1.5
MD_2	4,000	2,250	400	71~90	21~30	2,203±45	1.26±0.26	0.52±0.14	0.085±0.017	7.3±1.9
MD_3	4,000	2,500	600	111~130	31~40	2,200±84	1.42±0.52	0.46±0.11	0.074±0.019	8.1±2.7
MD_4	4,000	2,750	800	151~170	41~50	2,213±110	1.36±0.32	0.53±0.06	0.063±0.012	10.7±2.8
MD_5	5,000	2,000	400	111~130	41~50	2,230±105	1.46±0.47	0.47±0.18	0.084±0.021	8.6±3.4
MD_6	5,000	2,250	200	151~170	31~40	2,229±108	1.42±0.36	0.34±0.18	0.058±0.026	6.1±3.1
MD_7	5,000	2,500	800	31~50	21~30	2,151±73	1.56±0.38	0.81±0.13	0.104±0.009	8.4±1.5
MD_8	5,000	2,750	600	71~90	11~20	2,183±55	1.15±0.31	0.59±0.12	0.087±0.016	8.3±2.3
MD_9	6,000	2,000	600	151~170	21~30	2,180±72	1.35±0.25	0.44±0.14	0.062±0.022	8.5±2.3
MD_{10}	6,000	2,250	800	111~130	11~20	2,200±119	1.32±0.24	0.72±0.15	0.085±0.011	7.7±2.0
MD_{11}	6,000	2,500	200	71~90	41~50	2,204±118	1.07±0.20	0.50±0.18	0.083±0.021	8.5±2.5
MD_{12}	6,000	2,750	400	31~50	31~40	2,137±38	1.21±0.32	0.68±0.16	0.101±0.010	7.6±2.5
MD_{13}	7,000	2,000	800	71~90	31~40	2,209±79	1.35±0.27	0.75±0.15	0.097±0.013	8.4±2.1
MD_{14}	7,000	2,250	600	31~50	41~50	2,133±66	1.62±0.80	0.69±0.15	0.107±0.010	8.4±2.5
MD_{15}	7,000	2,500	400	151~170	11~20	2,166±104	1.42±0.31	0.39±0.10	0.073±0.019	7.9±2.4
MD_{16}	7,000	2,750	200	111~130	21~30	2,204±88	1.18±0.31	0.34±0.12	0.068±0.021	5.9±2.3

L_{AS1}: length of ancestral sequence 1. L_{AS12}: length of ancestral sequence 12. M_1: mutated bases per 1 branch length. $L_{I/D}$: length of bases inserted or deleted each time. $M_{I/D}$: number of bases mutated each time. L_{MSA}: length of multiple sequence alignment. R_{K2+I}: ratio of transition to transversion under K_{2+I} parameter model. $\bar{D}$: overall mean distance. $SE_{\bar{D}}$: standard error of the overall mean distance. TS_{ML}: topology score of the constructed ML tree. Data are presented as mean ± standard deviation (n=10).
