# Supplementary material for: Simulation of Chordate Intron Evolution Using Randomly Generated and Mutated Base Sequences
Source: Evol Bioinform Online. 2020 Jan 29;16:1176934320903108. doi: 10.1177/1176934320903108 (PMC6990610; doi:10.1177/1176934320903108)
Supplement: Table_S2_xyz299588fc8496b – Supplemental material for Simulation of Chordate Intron Evolution Using Randomly Generated and Mutated Base Sequences [file Table_S2_xyz299588fc8496b.pdf]

Table S2. Orthogonal test results of MI model

Test No.	Model parameters					Attributes of generated sequences				
	L_{AS1}	L_{AS12}	M_1	$L_{I/D}$	$M_{I/D}$	L_{MSA}	R_{K2+I}	$\bar{D}$	$SE_{\bar{D}}$	TS_{ML}
MI_1	10	110	200	31~50	11~20	2,099±61	1.57±0.16	1.14±0.11	0.094±0.013	8.9±2.2
MI_2	10	120	400	71~90	21~30	2,154±139	1.39±0.25	1.20±0.17	0.097±0.011	7.9±2.0
MI_3	10	130	600	111~130	31~40	2,093±36	1.67±0.61	1.11±0.18	0.099±0.008	7.6±1.4
MI_4	10	140	800	151~170	41~50	2,126±57	1.60±0.55	1.11±0.14	0.100±0.010	7.5±1.7
MI_5	20	110	400	111~130	41~50	2,104±57	1.63±0.23	1.20±0.12	0.093±0.007	5.7±1.7
MI_6	20	120	200	151~170	31~40	2,119±82	1.32±0.21	1.06±0.17	0.102±0.016	7.3±2.3
MI_7	20	130	800	31~50	21~30	2,116±41	1.29±0.21	1.16±0.10	0.094±0.013	7.5±2.0
MI_8	20	140	600	71~90	11~20	2,082±69	1.53±0.19	1.12±0.19	0.096±0.016	8.1±1.9
MI_9	30	110	600	151~170	21~30	2,089±79	1.34±0.33	1.18±0.16	0.096±0.011	6.6±2.7
MI_{10}	30	120	800	111~130	11~20	2,086±52	1.76±0.38	1.13±0.14	0.097±0.018	8.1±1.6
MI_{11}	30	130	200	71~90	41~50	2,105±62	1.70±0.36	1.20±0.16	0.091±0.013	6.9±2.6
MI_{12}	30	140	400	31~50	31~40	2,116±78	1.51±0.43	1.22±0.10	0.098±0.010	7.4±1.8
MI_{13}	40	110	800	71~90	31~40	2,119±103	1.70±0.27	1.17±0.13	0.092±0.011	7.3±2.1
MI_{14}	40	120	600	31~50	41~50	2,143±62	1.66±0.40	1.12±0.12	0.102±0.013	7.1±1.9
MI_{15}	40	130	400	151~170	11~20	2,118±59	1.49±0.36	1.15±0.06	0.096±0.006	6.9±1.5
MI_{16}	40	140	200	111~130	21~30	2,111±83	1.71±0.32	1.15±0.20	0.094±0.012	6.8±1.0

L_{AS1}: length of ancestral sequence 1. L_{AS12}: length of ancestral sequence 12. M_1: mutated bases per 1 branch length. $L_{I/D}$: length of bases inserted or deleted each time. $M_{I/D}$: number of bases mutated each time. L_{MSA}: length of multiple sequence alignment. R_{K2+I}: ratio of transition to transversion under K_{2+I} parameter model. $\bar{D}$: overall mean distance. $SE_{\bar{D}}$: standard error of the overall mean distance. TS_{ML}: topology score of the constructed ML tree. Data are presented as mean ± standard deviation (n=10).
